# 1020. A Novel Quality Control Solution for Gram Stain Through Integration of Deep Learning Vision Technology: A Proof-of-Concept

**DOI:** 10.1093/ofid/ofad500.051

**Published:** 2023-11-27

**Authors:** Jane Hata, Jessica Reed, Kyle Magee

**Affiliations:** Mayo Clinic Florida, Jacksonville, FL; Memorial Healthcare System, Hollywood, Florida; Mayo Clinic Florida, Jacksonville, FL

## Abstract

**Background:**

Gram stain (GS) is essential for preliminary bacterial identification. The lack of a standardized quality control method for GS is a significant drawback. By utilizing a deep convolutional neural network (CNN) design, we present a proof-of-concept for a novel solution to GS quality assurance. Using this approach, current manual GS, and potentially automated GS methods, may possess a standardized method of quality control.

**Methods:**

Twenty-nine GS from positive blood cultures encompassing the spectrum of GS quality (acceptable, over/under decolorization, smear thickness, stain artifact) were selected by two expert microbiologists.

A total of 197 images were obtained using a Cognex D-900 camera and Olympus BX-43 microscope at 1000X magnification. Images were classified using deep learning software (Cognex, ViDi Suite 4.1). Eighty-one images were classified as suboptimal and 116 were classified as optimal by expert review.

To address background variation due to manual GS, we implemented a screening classification tool to separate images into 5 distinct classes (pale, rose, orange, indigo, variegated). The training and test sets consisted of 100 and 97 images respectively.

Using a chaining technique, a second classification tool was added after the screening classification tool. The second tool further divided the 197 images into two dataset classes: optimal and suboptimal. The second tool used 105 images for training and 92 images for the test set. Model performance was compared to expert microbiology review, with GS verified by organism identification using MALDI-TOF.

**Results:**

Cognex ViDi Suite 4.1 could discriminate between optimal and suboptimal GS images. The chained classification tool was able to be trained with 100% recall and 100% accuracy, with a resulting F-score of 1.00.

Figure 1

**Figure:**
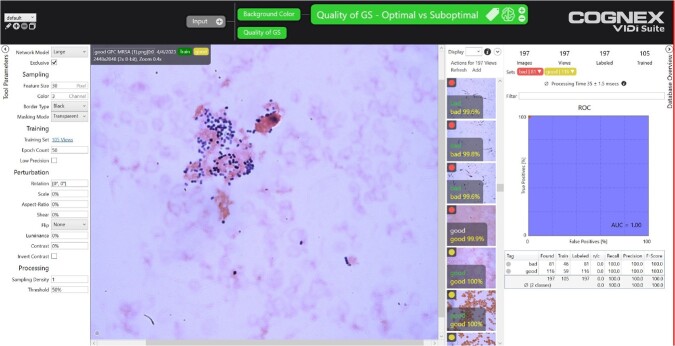

VisionPro GUI with optimal image (MRSA)

**Conclusion:**

Deep learning enabled image analysis is an effective method for sorting GS which are acceptable for further interpretation and suboptimal smears which require rework. Additional study is warranted to develop deep learning image analysis tools for the interpretation of GS on multiple specimen types and incorporate into automated GS systems.

**Disclosures:**

**Jane Hata, PhD , D(ABMM)**, Roche Molecular Systems: Grant/Research Support **Kyle Magee, MLS(ASCP)**, SeluxDx: Employee

